# Association between VEGF Gene Polymorphisms and In-Stent Restenosis after Coronary Intervention Treated with Bare Metal Stent

**DOI:** 10.1155/2017/9548612

**Published:** 2017-03-07

**Authors:** Zsolt Bagyura, Loretta Kiss, Kristóf Hirschberg, Balázs Berta, Gábor Széplaki, Árpád Lux, Zsolt Szelid, Pál Soós, Béla Merkely

**Affiliations:** ^1^Heart and Vascular Center, Semmelweis University, Városmajor Utca 68, Budapest 1122, Hungary; ^2^Department of Cardiology, University Hospital Heidelberg, Im Neuenheimer Feld 410, 69120 Heidelberg, Germany

## Abstract

*Background*. In-stent restenosis (ISR) is the gradual narrowing of the vessel lumen after coronary stent implantation due to the increase in vascular smooth muscle cell proliferation. Vascular endothelial growth factor (VEGF) protein plays an important role in this process. Our aim was to analyze the association of single nucleotide polymorphisms of the VEGF gene (rs2010963 and rs6999447) with the occurrence of ISR after coronary artery bare metal stent (BMS) implantation.* Methods*. 205 patients with a history of BMS implantation and a repeated coronarography were prospectively enrolled. Patients were assigned to diffuse restenosis group (*n* = 105) and control group (*n* = 100) and VEGF genotypes were determined.* Results*. Diffuse ISR was significantly more frequently observed in patients with homozygous normal genotype of rs2010963 polymorphism, and this polymorphism was independently associated with diffuse ISR.* Conclusions*. RS2010963 is associated with higher incidence of development of diffuse coronary ISR in patients treated with BMS implantation.

## 1. Introduction

In the era of balloon angioplasty without stent implantation in-stent restenosis (ISR) occurred in 40–50% [1]. Coronary stents were developed to lower the rate of early restenosis. However, ISR still occurs in 10–30% of the interventions with deployment of bare metal stents (BMS) and therefore still forms a clinically important problem [2]. Although new generation drug eluting stents (DES) further reduced ISR rate and need for repeat revascularization, BMS are still widely used. Moreover, recently Bønaa et al. found no significant difference between receivers of BMS and DES in the composite outcome of death from any cause or nonfatal spontaneous myocardial infarction [3].

After coronary stent implantation mechanical injuries of vessel wall result in vascular smooth muscle cell (VSMC) activation and proliferation and a phenotype change from contractile to proliferative and secretory phenotype [4, 5]. Histological analyses have revealed that vascular smooth muscle cells (VSMCs) represent the majority of neointima cells [6]. The neointima proliferation leads to narrowing of the coronary lumen [7].

Risk for restenosis is particularly high among patients with diabetes mellitus; this may be associated with metabolic alterations that promote endothelial dysfunction, accelerate intimal hyperplasia, and increase platelet aggregability and thrombogenicity [8]. There is evidence that gender itself (female) predisposes to restenosis [9] and some patients may have genetically higher risk [10, 11]. Genetic polymorphisms associated with high risk for restenosis include polymorphisms in genes coding for angiotensin II receptor type 1 [12], CD18 [13], interleukin-1 receptor antagonist [14], glycoprotein receptor IIIa [15], and mannose-binding lectin [16].

On invasive coronarography in-stent restenosis can be classified according to Mehran's classification to focal (Mehran I) and diffuse (Mehran II–IV) groups [17]. Former type is determined by local and procedural factors, while the latter shows significant relation with general, patient-related factors [18] such as diabetes or growth factors. Numerous studies of restenosis have indicated that transforming growth factor beta 1 (TGF-*β*1), platelet-derived growth factor beta (PDGFB), epidermal growth factor (EGF) [19], basic fibroblast growth factor (bFGF), and vascular endothelial growth factor (VEGF) play an important role in VSMC proliferation and migration to the tunica intima [20]. The rs699947 and rs2010963 polymorphisms are located in the 5′UTR region of the VEGF gene affecting the transcription and expression of the protein. The rs699947 polymorphism of the VEGF gene is associated with a higher risk of developing certain neoplastic diseases [21] and with the development of coronary collaterals in patients with coronary artery disease [22] or with susceptibility to coronary heart disease [23].

According to these findings the involvement of the VEGF in pathological processes leading to restenosis is known, but only one study has investigated the role of rs699947 gene polymorphisms in relation to restenosis [24], whereas the roles of functional polymorphism rs2010963 in restenosis have not yet been studied. The aim of this study was to analyze the association of VEGF's polymorphisms and the development of coronary in-stent restenosis after bare metal stent implantation.

## 2. Methods

### 2.1. Subjects, Interventions

205 patients with a history of percutaneous coronary intervention (PCI) and BMS implantation, who presented with nonacute or acute cardiac symptoms which warranted a repeat coronary angiogram, were prospectively enrolled between 2011 and 2013 in the Heart and Vascular Center, Semmelweis University. All patients received standard therapy according to the actual guidelines. ISR has been evaluated by experienced clinicians according to Mehran's classification and patients have been categorized to the diffuse restenosis group and control group (without diffuse restenosis).

### 2.2. Biological Samples and Genotyping

Genomic DNA was extracted from whole peripheral blood with a protease based technique (Flexigene DNA System, Qiagen, Hilden, Germany). Samples (1 mL) were added to a lysis buffer and were thoroughly mixed and centrifuged. After discarding the supernatant, samples were denaturized, and DNA was ethanol precipitated and reconstituted in the provided buffer. Samples were stored at −80°C. Estimation of the DNA yield and quality control was done by spectrophotometry and determination of the 260/280 absorption ratio (Nanodrop-2000, Thermo Scientific, Wilmington, USA), and the average yield was 96.0 *μ*g (range 25–370 *μ*g).

Determination of the alleles of the VEGF gene C−2578A (rs699947) was performed with quantitative real-time PCR (RT-qPCR) (StepOne Plus, Applied Biosystems). Predesigned primers were provided by Applied Biosystems (kit number: c___8311602_10). Reactions were performed according to the manufacturer's protocol and for each run parallel samples with positive controls were used.

Genotyping of G+405C (rs2010963) was performed by RT-qPCR and melting curve analysis using LightCycler (Roche GmbH, Penzberg, Germany). The following primers were used: 5′-CCAGAAACCTGAAATGAAGG-3′ in the forward direction and 5′-GGGCTCGGTGATTTAGC-3′ in the reverse direction, the probe sequences were 5′-LC640-TGG AAT TGG ATT CGC CAT TTT ATT TTT CTT gC-3′ and 5′-GAC CCA GCA CGG TCC CTC-FL. The PCR mix contained 1 *μ*L of the genomic DNA, 5 *μ*M of primers and probes, 1 *μ*L of LightCycler FastStart DNA Master HybProbe kit (Roche), and 2.5 mM MgCl^2^. The initial 10 min denaturation at 95°C was followed by 35 cycles, denaturation (95°C; 10 s), annealing (52–56–60°C; 15 s), and extension (72°C; 10 s), on LightCycler. Melting curve analysis was performed following the PCR and the* T*_*m*_ of the products was determined. The melting points (*T*_*m*_) were 59°C for C and 67°C for G alleles.

All assays were performed in 96-well arrays, and each plate contained controls. Genotyping of 10% of the samples was performed for quality control, with complete congruence. Genetic analysis was performed blinded to patient data, with the provided software. We established the genotypes for all patients. The genotype frequencies of two SNPs were all in agreement with the Hardy–Weinberg equilibrium test (*p* > 0.05).

### 2.3. Statistical Analyses

Data were collected in Microsoft Excel 2003 and were analyzed with SPSS 13.0 for Windows (SPSS, Chicago, USA) software. Data are presented as mean ± SD for continuous variables, or *n* (%) for categorical variables. Comparisons between two groups were performed using Student's* t*-test for continuous variables, whereas for continuous nonparametric variables Mann–Whitney* U* tests were performed. Categorical values were compared by using the chi-square test. Multivariate logistic regression has been performed with adjustment for clinical variables that reached a *p* value of <0.3 when comparing patients with and without significant diffuse ISR. The genotype frequency was tested for deviation from Hardy–Weinberg equilibrium by using Pearson's chi-square test. All analyses were performed two-tailed, and *p* < 0.05 was considered as significant.

## 3. Results

### 3.1. Patient Characteristics

The total number of 205 patients was involved in our study and categorized into diffuse restenosis and control group. The diffuse restenosis group (*n* = 105) included patients with significant diffuse ISR (Mehran II–IV) at recoronarography (73.3% men). Control group (*n* = 100) included patients with no or only focal restenosis (Mehran I) at recoronarography in the target bare metal stent (74.0% men).

Clinical baseline characteristics of the 205 patients are described in Table 1. The mean age (control: 66.5 (±10.2) years versus restenosis: 65.7 ± 9.8 years; *p* = 0.601) and distribution of genders (control: 74% male versus restenosis: 73.3% male; *p* = 0.914) did not differ significantly. About one-third of patients had diabetes mellitus (71 patients, 35%), mostly type 2. Most patients had hypertension and hyperlipidaemia; only 5.3% were not treated for hypertension and 9.1% were not on lipid-lowering therapy and had normal lipid levels. Obesity (body mass index (BMI) > 25) was present in 76.1% of patients. 21 (10.2%) cases had history of stroke or TIA and 31 (15.1%) cases had peripheral artery disease in the anamnesis; 22 (10.5%) of the patients were current smokers at the time of the second angiography. 10 patients had chronic renal failure and 6 patients had cardiogenic shock. The presence of all above listed factors except chronic renal failure did not differ significantly in the groups. The average time to repeated angiogram was 2.6 ± 2.7 years in the control group and 1.1 ± 1.4 years in the restenosis group (*p* < 0.001). According to this, in-stent restenosis occurred and caused symptoms earlier while controls had a longer asymptomatic period before recoronarography was performed.

### 3.2. Interventions

There was no significant difference in the indication of stent implantation between the two groups: acute coronary disease in 56.6% of the control group and 59.0% in the restenosis group. Total number of deployed stents was not significantly higher in restenosis group: 1.42 (0.75) versus 1.65 (0.97) (*p* = 0.056). Total stent length and stent diameter were not significantly different between the two groups (Table 1). One-tenth of the patients had multiple branches stented for the first intervention, 8 (8%) in the control group and 14 (13.3%) in the restenosis group (*p* = 0.004).

### 3.3. Genetic Analysis Results

Allele frequencies were similar between genders (rs2010963: 50.1%/41.7%/9.3% in men and 40.7%/50%/9.3% in women, (G/G, G/C, C/C) *p* = 0.454; rs699947: 25.5%/48.3%/26.2% in men and 15.4%/48.1%/36.5% in women, (A/A, A/C, C/C) *p* = 0.209). The genotype distribution conformed to Hardy–Weinberg equilibrium (*p* = 0.45 and *p* = 0.65, resp.). Genotype distributions for the groups of patient with no restenosis, focal restenosis, and diffuse restenosis are shown in Table 2. There was no statistically significant difference among these groups. For further analysis, we merged the focal restenosis group with the no-restenosis group as the focal in-stent restenosis is suspected to have different underlying pathomechanism compared to diffuse in-stent restenosis. Comparison of the genotype distribution of this control group to the diffuse in-stent restenosis group showed that diffuse in-stent restenosis was significantly less frequent in C/G and C/C genotypes (variant carrier) of rs2010963 polymorphism versus individuals with the G/G (homozygous normal) genotype (OR 0.56, *p* = 0.04). Restenosis frequency did not differ between the two groups for rs699947 polymorphism (Table 3).

### 3.4. Multivariate Analysis

Multivariate analysis adjusted for clinical variables (BMI, hypertension, smoking, chronic renal failure, average time to repeated angiogram, multiple branch stent deployment, total stent length, and total number of implanted stents) revealed that the homozygous normal (A/A) genotype of rs2010963 is related to higher risk of diffuse ISR. The rs699947 polymorphisms of VEGF gene are not associated with a risk of diffuse ISR (Table 4).

## 4. Discussion

In our nonrandomized prospective study, we found a significant association of rs2010963 VEGF polymorphism and the development of diffuse in-stent restenosis after coronary BMS implantation. This association was independent of certain clinical factors. VEGF is expressed by vascular endothelial cells, as well as additional cell types [25]. Association has been identified between VEGF polymorphisms and the risk of coronary artery disease [26], the development of collateral circulation in individuals with coronary artery disease [22]. VEGF contributes to mediating neovascularization of atherosclerosis plaques [27] and has been found to be associated with intimal thickening and thrombus development [28] and higher VEGF levels after PCI are related to restenosis after DES implantation [29].

Following PCI, a process similar to wound healing takes place in the affected coronary artery. VEGF has an important role directly in the progress of that endothelialization process and also it has an indirect effect on the inflammation cascade and VSMC proliferation and migration [30]. Accordingly, the endothelial stimulating effect of VEGF is essential for restoring the integrity of the vessel wall but it can also become the cause of restenosis through neointima hyperplasia [31]. Slight modifications of VEGF production or function due to gene polymorphisms can have remarkable consequences. Both rs699947 and rs2010963 are located in the promoter region (5′UTR) and known to influence the transcription of VEGF gene resulting in a lower serum level of the protein [32]. Rs2010963 (+405C>G) is probably a functional polymorphism, as serum VEGF levels in subjects with GG genotype have been found to be higher than in other genotypes [32].

Rs699947 has been previously associated with coronary artery disease [23]. It is noteworthy that Osadnik et al. [24] investigated the role of this polymorphism in development of angiographically significant ISR, but, in their study, focal and diffuse ISR was not distinguished. However, we compared patients with diffuse ISR to patients with no or focal ISR because diffuse ISR has different underlying mechanism compared to focal one, as the former is related to general factors, such as genotype, and the latter is rather related to procedural factors. Although rs699947 has been previously associated with coronary artery disease, our findings are in concordance with Osadnik et al.'s [24] observation as we found no significant association between diffuse ISR and rs699947.

Polymorphism rs2010963 was demonstrated to be associated with several disorders, such as diabetic retinopathy [33], diabetic nephropathy [34], metabolic syndrome [35], myocardial infarction [36], and impaired prognosis in patients with chronic heart failure [37]. VEGF rs2010963 SNPs have been associated with development of collateral circulation [22] and CAD [38]. In contrast, no association has been observed with HELLP syndrome (Hemolysis Elevated Liver enzymes Low Platelet count) according to Nagy et al. [39], although rs699947 genotype carriers had an increased risk of HELLP syndrome. Merlo et al. found significant association between VEGF levels and rs2010963 polymorphism but observed no association with carotid intima-media thickness [40]. Moreover, in a recent study [23] this SNP was not found to be associated with coronary artery disease. We found a significant association of rs2010963 VEGF polymorphism and the development of diffuse in-stent restenosis in our study population. The association remained present after adjusting for certain clinical factors in multivariate analysis. Particularly, we found that the association is independent of the presence of hypertension. As an influence of the polymorphism on hypertension has been described before [41], it is relevant that our observation implies an effect of the polymorphism on ISR without regard to hypertension. It is notable that only the rs2010963 showed association with diffuse in-stent restenosis even if both SNPs are known to affect the transcription and expression of* VEGF*. The explanation for that could be due to the fact that in a highly polymorphic gene such as VEGF no single SNP is responsible for the VEGF production, but, rather, the influence of multiple SNPs (i.e., haplotypes) is more likely. Additional SNPs residing on the same haplotypes (i.e., in linkage disequilibrium) could represent the causal or functional variants. The genetic variations of the gene need further study in order to refine the “at-risk” haplotype, which can then be used in a larger prospective study of patients [42].

## 5. Conclusions

In our nonrandomized prospective study, we found a significant association of rs2010963 VEGF polymorphism and the development of diffuse in-stent restenosis after coronary BMS implantation. This association was independent of certain clinical factors. We found no significant association between diffuse ISR and rs699947. Preventing restenosis is an important issue of coronary interventions that should start even before the procedure. Early recognition of the diseases and quick reaction usually mean that we face a less complicated lesion, therefore a lower restenosis rate. But there are some factors associated with ISR that we cannot influence, such as genetic polymorphisms. With the rapid development of genotyping technologies, examination of several polymorphisms may be accessible as routine diagnostics and therefore personalized risk assessment could be performed enabling better selection of patients for primary DES or bioabsorbable stent implantation.

## Figures and Tables

**Table 1:** Patient characteristics. Continuous variables are presented as mean (±SD). Dichotomous variables are presented as number of patients (percentage).

Variable	Control group (*n* = 100)	Restenosis group (*n* = 105)	*p* value
Age, years	66.5 (±10.2)	65.7 (±9.8)	0.601
Gender, male	74 (74%)	77 (73.3%)	0.914
Antihypertensive therapy	96 (97.0%)	98 (93.3%)	0.229
Lipid-lowering therapy	90 (91.8%)	95 (90.5%)	0.733
Diabetes mellitus	34 (35.1%)	37 (36.6%)	0.816
Obesity, BMI > 25	79 (82.3%)	77 (74.8%)	0.197
BMI	28.0 (±3.8)	28.1 (±4.4)	0.817
Smoking	13 (13.0%)	8 (7.6%)	0.204
Peripheral vascular disease	13 (13%)	18 (17.1%)	0.396
Chronic renal failure	7 (7%)	3 (2.9%)	0.123
Maximal CKMB level	114.8 (±102.3)	95.3 (±140.3)	0.322
Acute coronary disease	56 (56.6%)	62 (59.0%)	0.720
Angina pectoris	40 (40%)	37 (35.2%)	0.482
Cardiogenic shock (Killip IV)	2 (2%)	4 (3.8%)	0.527
Multi-branch stented	8 (8.0%)	14 (13.3%)	0.004
Total number of stents	1.42 (±0.75)	1.65 (±0.97)	0.056
Total stent length in mm	30.7 (20.7)	38.9 (25.5)	0.119
Stent diameter in mm	3.1 (0.4)	3.2 (2.6)	0.614
Ejection fraction	52.5 (11.2)	52.8 (10)	0.864
Average time to repeated angiogram (years)	2.65 (±2.72)	1.12 (±1.43)	<0.001

**Table 2:** Genotype distribution of no-restenosis group and focal and diffuse restenosis groups; G/G versus G/C + C/C and A/A versus A/C + C/C, chi-square.

Polymorphism	Genotype	No-restenosis group *n* (%)	Focal restenosis group (%)	Diffuse restenosis group *n* (%)	*p*
rs2010963	Normal (G/G carriers)	28 (40%)	13 (43.3%)	58 (55.2%)	0.119
	Variant (G/C + C/C carriers)	42 (60%)	17 (56.7%)	47 (44.8%)	
rs699947	Normal (A/A carriers)	13 (18.6%)	8 (26.7)	28 (26.7%)	0.436
	Variant (A/C + C/C carriers)	57 (81.4%)	22 (73.3%)	77 (73.3%)	

**Table 3:** Genotype distribution of control group (no-restenosis + focal restenosis) versus diffuse restenosis group; G/G versus G/C + C/C and A/A versus A/C + C/C, chi-square.

Polymorphism	Genotype	Control group *n* (%)	Diffuse restenosis group *n* (%)	*p*
rs2010963	Normal (G/G carriers)	41 (41%)	58 (55.2%)	0.041
	Variant (G/C + C/C carriers)	59 (59%)	47 (44.8%)	
rs699947	Normal (A/A carriers)	21 (21%)	28 (26.7%)	0.342
	Variant (A/C + C/C carriers)	79 (79%)	77 (73.7%)	

**Table 4:** Results of the multivariate logistic regression analyses with adjustment for risk factors and VEGF rs2010963 variant genotype (G/C + C/C) with in-stent restenosis as a dependent variable. Hosmer–Lemeshow test *p* = 0.139.

Risk factor	OR	CI		*p*
		Lower	Upper	
Hypertension or antihypertensive therapy	0.45	0.043	4.692	0.503
BMI	1.05	0.906	1.235	0.478
Smoking	0.493	0.088	2.757	0.42
Chronic renal failure	0.754	0.034	16.807	0.859
Multi-branch stented	0.982	0.217	14.368	0.596
Total number of stents	1.441	0.421	4.939	0.561
Average time to repeated angiogram	0.226	0.756	0.413	0.004
Total stent length	1.002	0.958	1.049	0.992
VEGF rs2010963	0.754	0.034	0.535	0.003

## Abbreviations

BMI: Body mass index
BMS: Bare metal stent
CAD: Coronary artery disease
CI: Confidence interval
CKMB: Creatine kinase MB isoform
DES: Drug eluting stent
DNA: Deoxyribonucleic acid
ISR: In-stent restenosis
OR: Odds ratio
PCI: Percutaneous coronary intervention
SD: Standard deviation
SNP: Single nucleotide polymorphism
VEGF: Vascular endothelial growth factor.
